# Non-Contact Thermal and Acoustic Sensors with Embedded Artificial Intelligence for Point-of-Care Diagnostics

**DOI:** 10.3390/s24010129

**Published:** 2023-12-26

**Authors:** Luís Rodríguez-Cobo, Luís Reyes-Gonzalez, José Francisco Algorri, Sara Díez-del-Valle Garzón, Roberto García-García, José Miguel López-Higuera, Adolfo Cobo

**Affiliations:** 1 CIBER de Bioingeniería, Biomateriales y Nanomedicina, Instituto de Salud Carlos III, 28029 Madrid, Spain; luis.rodriguez@unican.es (L.R.-C.); miguel.lopezhiguera@unican.es (J.M.L.-H.); adolfo.cobo@unican.es (A.C.); 2 Photonics Engineering Group, University of Cantabria, 39005 Santander, Spain; luisrafael.reyes@unican.es; 3 Instituto de Investigación Sanitaria Valdecilla (IDIVAL), 39011 Santander, Spain; 4 Ambar Telecomunicaciones S.L., 39011 Santander, Spain; sdiez@ambar.es (S.D.-d.-V.G.); rgarcia@ambar.es (R.G.-G.); 5 Centro de Innovación de Servicios Gestionados Avanzados (CiSGA) S.L., 39011 Santander, Spain

**Keywords:** thermal, acoustic, sensors, remote, low-cost hardware, neural networks

## Abstract

This work involves exploring non-invasive sensor technologies for data collection and preprocessing, specifically focusing on novel thermal calibration methods and assessing low-cost infrared radiation sensors for facial temperature analysis. Additionally, it investigates innovative approaches to analyzing acoustic signals for quantifying coughing episodes. The research integrates diverse data capture technologies to analyze them collectively, considering their temporal evolution and physical attributes, aiming to extract statistically significant relationships among various variables for valuable insights. The study delineates two distinct aspects: cough detection employing a microphone and a neural network, and thermal sensors employing a calibration curve to refine their output values, reducing errors within a specified temperature range. Regarding control units, the initial implementation with an ESP32 transitioned to a Raspberry Pi model 3B+ due to neural network integration issues. A comprehensive testing is conducted for both fever and cough detection, ensuring robustness and accuracy in each scenario. The subsequent work involves practical experimentation and interoperability tests, validating the proof of concept for each system component. Furthermore, this work assesses the technical specifications of the prototype developed in the preceding tasks. Real-time testing is performed for each symptom to evaluate the system’s effectiveness. This research contributes to the advancement of non-invasive sensor technologies, with implications for healthcare applications such as remote health monitoring and early disease detection.

## 1. Introduction

Remote patient diagnostics is a rapidly growing field, with significant advancements in sensor technologies and artificial intelligence algorithms. These advancements have revolutionized the way we monitor and diagnose various health conditions, making healthcare more accessible and efficient. One of the key health indicators that can signal the presence of various diseases is body temperature [1]. It is essential for the human body to maintain an appropriate body temperature level for optimal physiological functioning. Depending on the measurement site, the temperature can vary for healthy individuals. For instance, oral temperature typically ranges between 33.2 and 38.2 °C, rectal temperature between 34.4 and 37.8 °C, tympanic temperature between 35.4 and 37.8 °C, and axillary temperature between 35.5 and 37.0 °C [2]. The importance of monitoring body temperature has been recognized since ancient times, leading to the invention of various devices and techniques for temperature measurement. However, it was not until the 18th century when the first mercury-in-glass thermometer was developed by Gabriel Fahrenheit [3]. This invention marked a significant milestone in the history of medical thermometry. However, traditional contact thermometers have certain limitations that can affect the accuracy and safety of temperature monitoring [4]. For instance, they require physical contact with the body, which can be uncomfortable and potentially spread infectious diseases. The advent of infrared technology in the mid-20th century introduced non-contact temperature sensing. This technology detects infrared radiation emitted by objects to determine their temperature. Non-contact infrared thermometers (NCITs) and thermal scanners employ this principle to measure skin surface temperature without physical contact. This non-contact approach makes it possible to measure the temperature at a certain distance, reducing the risk of infections by air transmission [5]. With the onset of the COVID-19 pandemic, non-contact temperature screening methods have been extensively used in crowded places like airports, markets, stations, and hospitals [6]. The pandemic underscored the need for quick and accurate fever detection to curb virus spread. This highlighted the importance of non-contact temperature measurement technologies in public health and disease control.

In the fight against COVID-19-induced pneumonia, another important application is cough detection. Recent studies reveal that audio sounds produced by the respiratory system, such as coughing, can be analyzed to determine whether a patient is infected with COVID-19 [7]. This is a significant breakthrough as it allows for non-invasive and rapid screening of potential COVID-19 cases. Cough analysis has been utilized for COVID-19 screening and diagnosis. Machine learning techniques, which involve algorithms that improve automatically through experience and by the use of data, can provide valuable insights that aid in the development of a diagnostic tool [8]. However, to achieve this, a substantial amount of cough data from both COVID-19 and non-COVID-19 patients is needed. These data serve as the foundation for training the machine learning models, allowing them to learn the distinguishing features of COVID-19 coughs. For example, in [9], a database called Coswara is proposed, which contains respiratory sounds like coughs, breaths, and voices. This database serves as a valuable resource for researchers working on COVID-19 cough detection. Recent experiments have been conducted to screen for COVID-19 using acoustic features extracted from these respiratory sounds. In [10], a recurrent neural network (RNN), specifically the long short-term memory (LSTM) architecture, is used to extract six speech features from a collected dataset. They used 70% of the data for training and 30% for testing. The model achieved the highest accuracy with breathing sounds (98.2%), followed by cough sounds (97%), and voice analysis (88.2%). This demonstrates the potential of RNNs in analyzing respiratory sounds for COVID-19 detection. In [11], a smartphone application for COVID-19 detection is proposed based on cough diagnosis. The application, called AI4COVID-19, operates by sending three 3 s cough sounds to an AI engine running in the cloud and providing a result within two minutes. Due to the scarcity of COVID-19 cough training data, the authors applied transfer learning using the ESC-50 dataset (Piczak 2015), which contains 50 classes of cough and non-cough sounds recorded using a smartphone. The results showed an overall accuracy of 95.60%, a sensitivity of 96.01%, a specificity of 95.19%, and precision of 95.22%. This demonstrates the effectiveness of the application in detecting COVID-19 based on cough sounds. Another interesting work involves the use of the statistics of the sound signals, analysis in the frequency domain, and Mel-frequency cepstral coefficients (MFCCs) to reveal whether a user has COVID-19 or not. Test results show, amazingly, an accuracy of over 97% with a convolutional neural network (CNN) classifier and more than 85% on k-nearest neighbors (kNN) with optimized features. This highlights the potential of using advanced signal processing techniques and machine learning models in COVID-19 detection. Other works can be found in several review articles about this topic [7,12]. These articles provide comprehensive overviews of the current state-of-the-art in COVID-19 detection using cough sounds.

In summary, the field of remote patient diagnostics is experiencing rapid advancements thanks to improvements in non-contact thermal and acoustic sensors, low-cost hardware, and neural networks. These technologies have the potential to revolutionize healthcare, making it more accessible and efficient. However, challenges still need to be addressed to improve the accuracy and reliability of these technologies. In this work, we propose low-cost, non-invasive sensor technologies for data collection and preprocessing, explicitly focusing on novel methods of thermal calibration and the assessment of low-cost infrared radiation sensors for facial temperature analysis. Additionally, we investigate innovative approaches to analyzing acoustic signals for quantifying coughing episodes. Our research integrates diverse data capture technologies to analyze them collectively, considering their temporal evolution and physical attributes. Our aim is to extract statistically significant relationships among various variables for valuable insights. This integrated approach allows us to leverage the strengths of each technology, leading to more accurate and reliable diagnostic tools.

## 2. Materials and Methods

**Figure 1:**
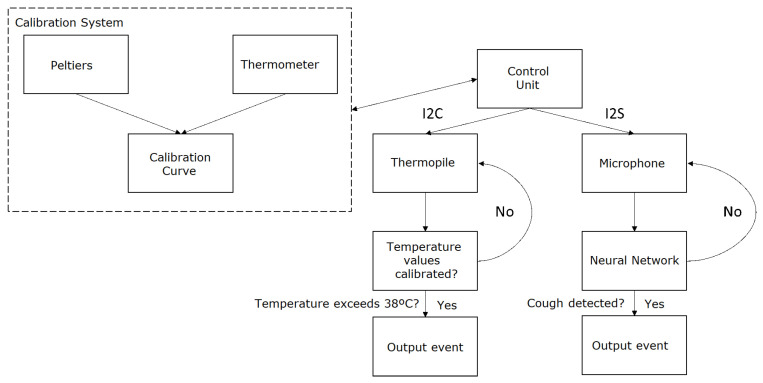
Proposed system diagram.

The proposed system, designed to meet the system specifications, is illustrated in Figure 1. The system design clearly differentiates between the two main specifications of the system: cough detection and temperature measurement. Cough detection is achieved using a microphone coupled with a neural network. The microphone captures the sound, which is then processed by the neural network to identify cough sounds. The neural network is trained on a large dataset of various cough and non-cough sounds, enabling it to accurately distinguish between the two. This allows for real-time cough detection, which is crucial for monitoring the health status of individuals in various settings. On the other hand, the thermopile sensor is used for non-contact temperature measurement. The thermopile adjusts its output values through a calibration process, which involves creating a straight line between the temperatures of maximum interest. This calibration process significantly reduces the error within a temperature range defined by the calibration points, resulting in more accurate temperature readings. As for the control units, the system was initially based on an ESP32 microcontroller. The ESP32 was chosen for its high performance, low power consumption, and advanced features such as Wi-Fi and Bluetooth connectivity. However, due to implementation issues with the neural network, the entire system was migrated to a Raspberry Pi model 3B+. The Raspberry Pi model 3B+ offers more computational power and flexibility, making it better suited for running complex neural network models. Furthermore, it provides a wide range of connectivity options, including Ethernet, Wi-Fi, and Bluetooth, and supports a variety of operating systems, offering more flexibility for software development.

### 2.1. Hardware and Software Involved

The research utilizes the ESP32 and Raspberry Pi model 3B+ as its core hardware components.

The ESP32, a solution for Wi-Fi and Bluetooth IoT applications, features the ESPRESSIF-ESP32-DOWDQ6 chipset and a 240 MHz Xtensa LX6 microprocessor, enabling smooth operation. It comes equipped with up to 32 MB of memory for firmware and data storage, a 1.3-inch IPS display, a 2-megapixel camera, and a low power profile suitable for battery-powered applications. The device’s compact dimensions make it ideal for space-constrained environments. It also includes an MSM261S4030H0 microphone, Micro SD external storage, and an IP5306 power management unit, offering versatile functionality while meeting international regulatory standards for wireless connectivity.

The Raspberry Pi model 3B+ is a single-board computer powered by the Broadcom BCM2837B0 chipset with a Cortex-A53 64-bit SoC. It provides significant computational power with a 1.4 GHz clock speed and 1 GB LPDDR2 SDRAM. Storage is managed via a micro SD card, offering flexibility. The Raspberry Pi operates within a wide temperature range and is compact in size, making it suitable for various applications. It includes an INMP441 microphone, Micro SD external storage, and robust security and encryption features for secure wireless communication. The device operates on the Raspbian operating system, optimized for Raspberry Pi hardware.

Both devices are characterized by their efficient processing capabilities, low power consumption, compact size, and comprehensive security features, making them well-suited for the study’s requirements in remote health monitoring.

### 2.2. Thermal Sensor

The task at hand involved the design of non-invasive temperature sensors. This was addressed by testing various non-contact thermal sensors to determine the most suitable for our application. The different sensors tested included AMG8833 [13], HTPA80x64d [14], and Melexis MLX90640 [15].

Initially, these sensors were used with a low-cost microcontroller, the ESP32. This microcontroller was chosen due to its compact size, low power consumption, and integrated Bluetooth and Wi-Fi capabilities, making it ideal for the purpose of this task.

To assess the suitability of these sensors for temperature monitoring, they were connected via I2C to an ESP32 microcontroller. The microcontroller read data from the sensors and transmitted them via a serial port. These data were further processed by a Python program, which represented the temperature information in a heatmap. This visual representation allowed for a more intuitive understanding of the temperature distribution.

Various tests were conducted with the different sensors, leading to diverse outcomes:

- AMG8833: This sensor, featuring an 8 × 8 two-dimensional infrared array, exhibited an accuracy of ±2.5 °C. However, it was ruled out due to its lack of precision. Its relatively large pixels led to high thermal variability when encompassing objects at different temperatures within the same pixel.
- HTPA 80 × 64 L4.8: This infrared thermopile sensor, with an 80 × 64 resolution, 90 × 70° field of view, 14.6 mm target length, 20 mm target diameter, and 0.8 mm focal length was dismissed due to stability issues over time. Despite its high resolution, the sensor was not reliable for long-term use.
- Melexis MLX90640: This infrared thermopile sensor, with a 32 × 24 resolution and a 110° × 75° field of view was selected for its precision and stability. The smaller pixel size resulted in improved accuracy when capturing from the same distance as the AMG8833.

**Figure 2:**
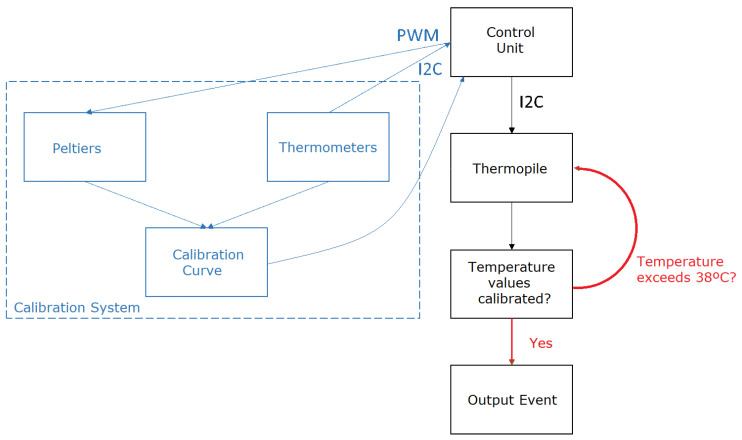
Diagram of the thermal system.

A schematic of the thermal system is provided in Figure 2, distinguishing between the calibration system (in blue), temperature output (in red), and basic control system (in black). This schematic provides a clear overview of the system’s architecture and the interaction between its components.

The calibration system plays a crucial role in correcting the thermopile error within the temperature range of interest. It comprises two Peltier elements and two temperature sensors that regulate their temperatures. The Peltiers are set to 33 °C and 38 °C, with a PID control system implemented by the microcontroller to maintain these temperatures. The PID control adjusts the duty cycle of a pulse-width modulation (PWM) signal controlling the Peltier. A Peltier is an active solid-state heat pump that transfers heat from one side of the device to the other, consuming electrical energy depending on the current direction.

The operation of this calibration system involves the following steps for each captured sample: the thermopile captures the temperatures of the Peltiers, and the temperature obtained from the temperature sensors is used to create a calibration curve that corrects the thermopile error. This calibration process ensures that the temperature readings from the thermopile are accurate and reliable.

The temperature sensors, responsible for providing feedback to the calibration system through PID control, include:

- BMP085: A pressure and temperature sensor with an accuracy of ±2 °C (±1 °C around 25 °C). This sensor provides reliable temperature readings within the specified accuracy range.
- MCP9800 [16]: A temperature sensor with an accuracy of ±1 °C. This sensor offers higher accuracy compared to the BMP085, making it suitable for applications that require precise temperature measurements.

**Figure 3:**
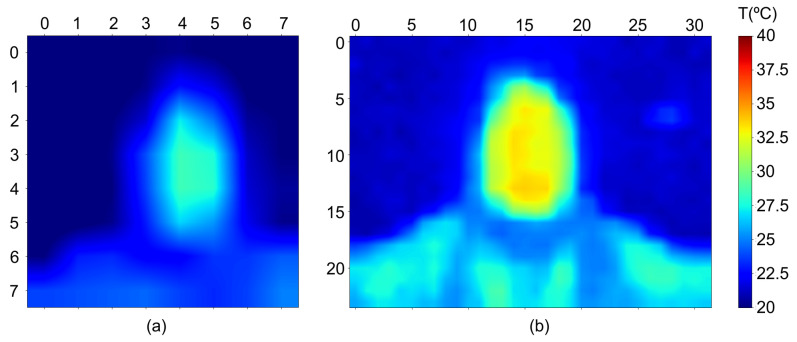
Captured thermographic images with (**a**) sensor AMG8833 and (**b**) sensor MLX90640.

Some results of the obtained thermographic images are shown in Figure 3. Two different sensors were used to avoid conflicts in the I2C addresses for the thermometers within the temperature range of interest. The Peltiers used were the TEC1-12706 model, operating at 12 V with a maximum current of 5.8 A (60 W) and measuring 40 × 40 × 3.8 mm. These Peltiers were chosen for their optimal performance characteristics and suitable dimensions for our application.

As mentioned earlier, the thermopile is calibrated using two Peltiers set at temperatures close to those of interest, specifically 33 °C and 38 °C. The Peltiers serve as reference points for the thermopile, providing known temperatures for calibration. To ensure effective calibration, the Peltiers need to be positioned at the same distance from the thermopile as from the patient’s face. This is to ensure that the thermopile reads the same temperature distribution as it would when measuring a patient’s temperature.

The extremes of the field of view should be avoided to prevent optical aberrations, which could distort the temperature readings. The calibration process unfolds as follows: the system is initiated with the Peltiers at room temperature, and the Peltiers are heated until they reach the set temperatures. During this phase, it is important not to move thermal objects to different temperatures within the thermopile’s field of view, as the Peltier positioning self-detection relies on temperature fluctuations.

Within the thermopile’s field of view, the point with the highest temperature is identified, excluding areas where the Peltiers are located. This point is considered the patient. A calibration curve is created using the temperature of the Peltiers, obtained from I2C thermometers, and the temperature measured by the thermopile. This process is performed for each measurement. The average temperature of the pixel identified as the patient and its eight adjacent pixels is determined to enhance the system’s robustness. This temperature is calibrated based on the calibration curve to minimize error.

A test system consisting of an 11 × 11 cm aluminum plate simulating a patient’s head was developed for validation tests. This plate is covered with temperature-conductive material but insulated from electricity. It is equipped with a Peltier and a thermometer to control the temperature, which a microcontroller regulates. The square area of 11 × 11 cm is divided into nine parts to verify the temperature distribution and validate the method.

The DAQ970A [17] data acquisition system was used for this validation, with the temperature sensors RTD PT100 [18] employed to measure temperature variations at different points on the aluminum plate. Based on these results, the system was validated as a testing tool for the prototype.

**Figure 4:**
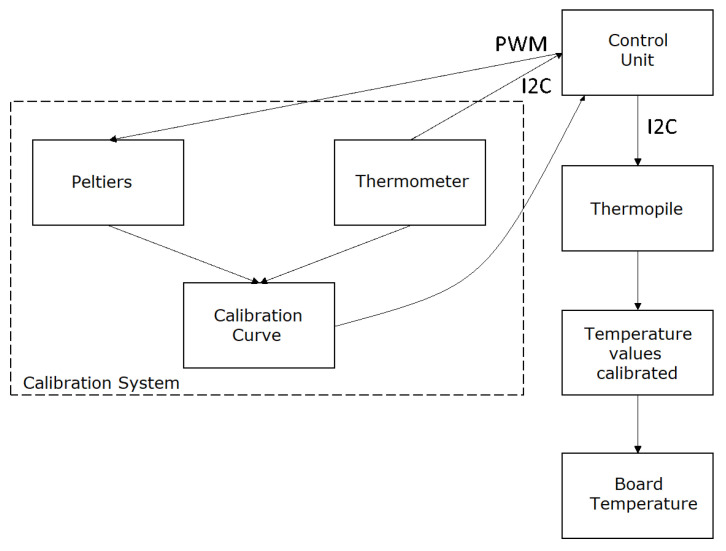
Validation test diagram.

The scheme followed for these validation tests is presented in Figure 4.

The only difference between the final system and this validation system is that the validation system provides a temperature value every second, which is necessary for system validation. Since the validation system has already been proven to be an effective tool for demonstrating the thermal system’s functionality, the next step involves taking measurements with the thermal system and the test tool to assess its accuracy.

The process for capturing various measurements involved:

1. Initiating the system, allowing it to calibrate with the Peltiers set at 33 °C and 38 °C, which takes approximately 2 min. This step ensures that the system is ready for accurate temperature measurements.
2. Introducing the validation system to a set temperature. This step involves adjusting the temperature of the validation system to a specific value for testing.
3. Recording the values read by the thermometer of the validation system (validation temperature) and the temperature measured by the thermopile (measured temperature). This step involves comparing the actual temperature with the temperature measured by the thermopile.

The obtained values and their errors are shown in Table 1. These results indicate an average error of 0.28 °C within the measurement range of 36.5 °C to 38.5 °C. This level of accuracy is acceptable for most medical applications, demonstrating the effectiveness of the thermal sensor system.

### 2.3. Acoustic Sensor

The development of the cough sensor involved the selection of an I2S microphone due to the necessity for continuous sound recording for subsequent analysis. This continuous recording is crucial for real-time monitoring and immediate response to cough detection. Two options were considered for this purpose: the SPH0645LM4H and the INMP441. The INMP441 was ultimately chosen due to its cost-effectiveness and availability in the market.

This microphone will continuously capture sound in the background while the device is active, generating an alert message when it detects a cough. This real-time alert system can be beneficial for immediate medical response and timely treatment. The cough detection will be carried out using deep learning, a subset of machine learning that imitates the workings of the human brain in processing data for use in decision making.

Three approaches were considered for implementing deep learning:

- Keras: a Python-based neural network library. This approach was dismissed because it is based on a high-level language and is difficult to implement on a microcontroller like ESP32, which requires more low-level programming.
- Pytorch: a machine learning library used for various applications, including natural language processing. Like Keras, it is also high-level, so this option was discarded due to similar reasons.
- TensorFlow: a machine learning library capable of building and training neural networks. It also has a tool for use in mobile and IoT devices, TensorFlow Lite, which allows training a model and exporting it for implementation on a mobile or IoT device. This feature makes TensorFlow suitable for our application.

**Figure 5:**
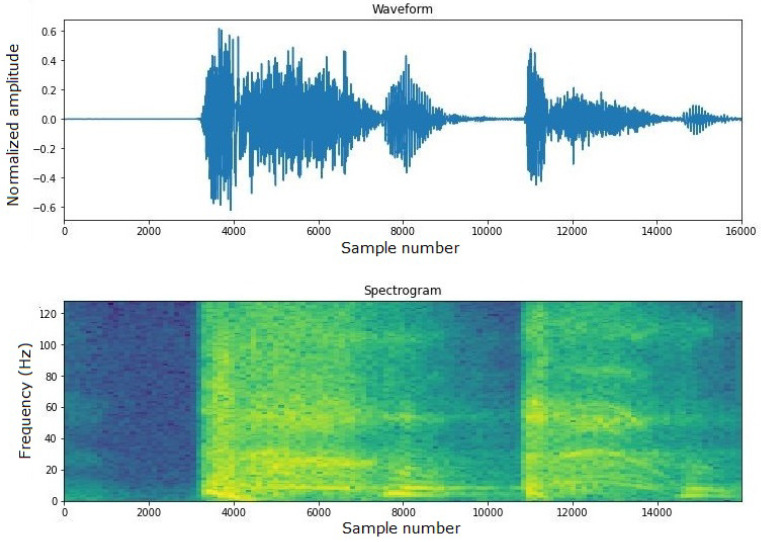
Example of cough spectrogram.

For these reasons, TensorFlow was chosen. In all three trained models, audio input is provided in .wav format, with a 16 kHz bitrate, a 1 s audio clip duration per sample, and a 30 ms window size. This standardization of input format ensures consistency in data processing and analysis. As mentioned earlier, model 3 is fed with the spectrogram of the audio input (see an example in Figure 5). The spectrogram provides a visual representation of the spectrum of frequencies in the audio signal as they vary with time, which can be useful for identifying unique patterns in the cough sound.

The dataset was obtained by mixing various datasets from platforms like Kaggle. Kaggle is a website where public and private challenges are published for various tasks. Due to the current era we are in, several cough detection challenges were found on Kaggle, making it easy to obtain a large number of samples. This diverse dataset enhances the robustness of the model by exposing it to a variety of cough sounds under different conditions.

The main problem with these samples was the lack of correlation between the specifications needed by the model and the audio samples. The main changes included converting the format to .wav, changing the bit rate for each sample, and adjusting the duration, as the audio samples were typically longer than 3 s. These modifications were necessary to ensure that the input data were compatible with the model’s requirements.

In the end, a robust and diverse dataset of around 3000 different samples (approximately 50% split between men and women) was obtained, with 70% used for training, 20% for validation, and 10% for testing. This distribution ensured that the model was exposed to a wide variety of data, enhancing its ability to generalize and perform well on unseen data. The samples were randomly assigned to each of the three groups to avoid any bias in the distribution of data.

**Figure 6:**
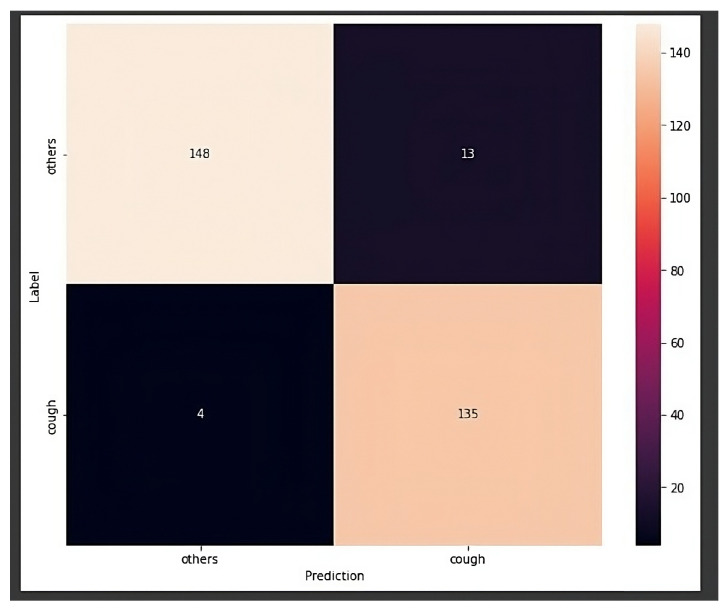
Confusion matrix for the chosen model.

Once the machine learning library and the created dataset were chosen, three different models were generated:

- Model 1: Initially, the Mobilnetv1 architecture was used in conjunction with YAMNet, a deep neural network capable of predicting 521 different audio classes trained on the AudioSet-YouTube corpus dataset. Transfer learning was applied to use it for cough recognition. However, the model size when converted to TFLite (3.85 MB) made it unsuitable due to the limited flash memory of the ESP32 microcontroller. This model achieved an accuracy of approximately 90% on the test set.
- Model 2: In the second model, a simpler neural network was used to avoid the size problem when using it on the ESP32. Once again, transfer learning was applied by adding the cough class to the original dataset. This model had a size of 15 kB and achieved an accuracy of approximately 88% on the test set. Due to discrepancies between the algorithms used by TensorFlow in the desktop application for training the network and those implemented by the TensorFlowLite library on the ESP32, it was decided to migrate the system to a Raspberry Pi 3B+. The memory size issue of the ESP32’s flash memory was also considered.
- Model 3: By using the Raspberry Pi, the first model with a size of 3.95 MB could have been used. However, a new model was created with the input format being the audio’s spectrograms rather than the raw audio collected from the microphone. This change in input format resulted in a more accurate and robust model, albeit at a slightly reduced speed. However, it was fast enough to work in real time. The confusion matrix, which compares the data predicted by the neural network with its predefined label, achieved over 94% accuracy for 300 audio samples, as shown in Figure 6.

Table 2 summarizes the sizes and accuracy of the different models. Comparing these models, it is evident that model 3 is the most accurate and optimized, as it has a smaller size.

The chosen model consists of a simple convolutional neural network, with input as the spectrograms directly derived from the input audio. The model also includes two layers that enhance the speed and accuracy of the trained model. A first layer (resizing layer) changes the size of the spectrogram from (129,160) to (129,124). The second layer (normalization layer) normalizes each pixel of the spectrogram based on the mean and standard deviation.

Testing on the neural network was performed after the training phase, as is customary. As mentioned earlier, the dataset was divided into three parts: 70% for training, 10% for testing, and 20% for validation. The validation dataset was the same for all three methods, allowing for a comparison of how each method responds to the same inputs. The testing dataset was used to check the accuracy of the neural network in labeling unknown inputs, and the training dataset was, as the name implies, used to train the neural network.

**Figure 7:**
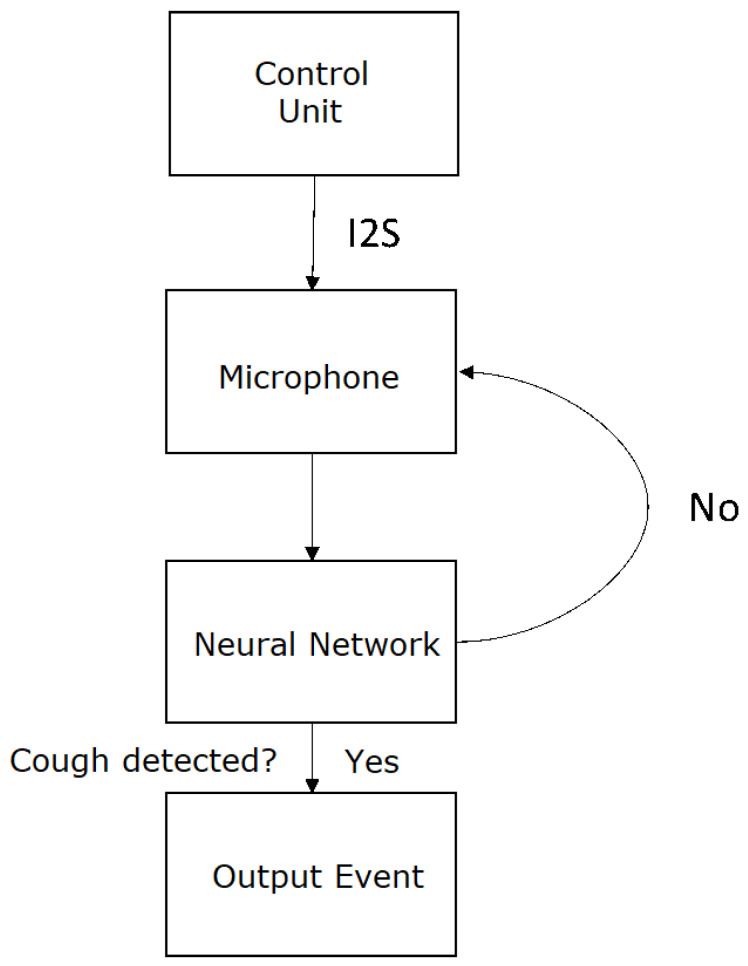
Flow diagram of cough detection system.

The neural network that yielded the best results in this test was model 3, where the input was the audio spectrogram, resulting in an accuracy of 94%. The flow diagram of cough detection system can be found in Figure 7.

Once the correct functioning of the system on the PC was verified, an attempt was made to replicate this behavior on the Raspberry Pi by inferring on the test dataset to emulate the 94% accuracy. This was successfully achieved, and the transition to real-world simulation and testing was carried out. Several tests were conducted in the laboratory by integrating the complete system and simulating coughing on the microphone. After the initial tests in the laboratory with the complete system in place, certain limitations of the model in correctly detecting coughs in a real-world simulation environment were observed, in contrast to the results obtained on pre-recorded videos. The main problems with the model occurred with dry coughs and with words that included the phoneme “S”, in which cases the model predicted coughs and had a severe limitation in detecting short coughs. To address the issue more effectively, an attempt was made to filter out silence correctly. A function was applied that resulted in the average value of the audio signal. However, it was observed that this filtering was incorrect, causing the model to predict any kind of audio. This failure in filtering was due, among other things, to the fact that this filtering occurred after the normalization of the input audio signal, which reduced the mean value of the audio signal, rendering the filter ineffective for detection.

In a subsequent step, it was decided to create a much larger dataset than the previous one, on the order of 20k samples evenly distributed between the cough class and the non-cough class. Training, although much more time consuming than in previous cases, provided minimal advantages, especially in detecting short coughs. The next decision was to vary the duration of the input audio for training. It was observed that when coughing twice in succession, the model only detected it as a single cough. Whereas when coughing only once, it did not detect it at all. It was thought that this occurred due to the breaks in the streaming audio, which caused the cough to fall into different windows, making it challenging to detect. As a result, a dataset of 20k samples was generated for 2 s per audio. Naturally, due to the same set of files but with each file lasting twice as long, the number of samples was half that of the previous test, around 10k. To perform the training, preprocessing of the obtained audio was necessary. The main features of these audios were:

- 2 s in duration;
- Mono-channel;
- 16 bits;
- 16 kHz bit rate.

**Figure 8:**
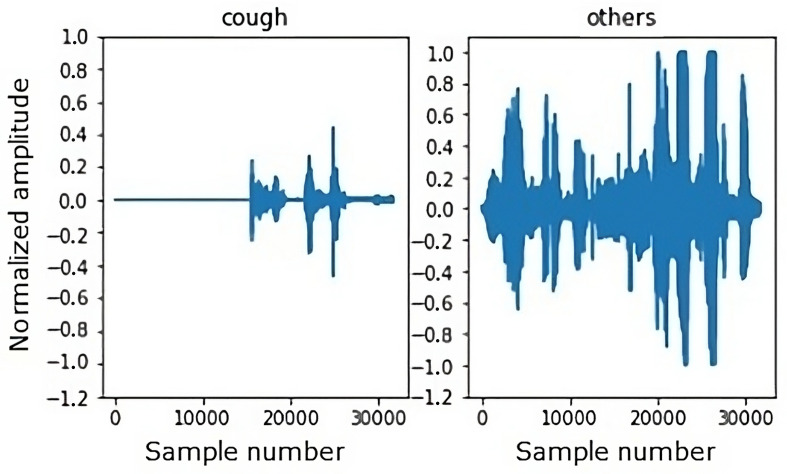
Example of a 2 s duration waveform.

In Figure 8, examples of audio signals for the cough and non-cough classes with a 2 s duration can be found.

The main difference observed between the audios in these datasets and those used in previous models was the number of samples. This was naturally due to the new duration of the audios. While they had previously contained 16k samples, they now had 32k. After obtaining audio samples with the specified characteristics, the audio signal was normalized, and it was converted to the frequency domain to obtain its spectrogram. This spectrogram would serve as input to the model for training and detection. The main variation in the sample compared to the previous models used was the size of the spectrogram, creating a sample of size 129,246. The results were significantly better compared to previous tests, especially in detecting short coughs. However, it was decided to perform one last test, further enriching the dataset by splitting it into several new classes.

Considering that the final results of the model executed from the Raspberry Pi did not meet the expected results, new approaches were considered to address the problem. After several new ideas were considered, it was decided to enrich the dataset with three new classes (music, conversation, and ambient noise) in addition to the previous classes (cough and non-cough). Recognizing that varying the audio duration helped to achieve better results in cough detection but did not address issues with dry coughs, an attempt was made to further differentiate each class. Three new classes were added to the existing ones:

- Ambient Noise. This class used audio obtained from various YouTube videos simulating ambient noise in an office, an operating room, and a hospital room.
- Music. This class used audio extracted from different types of music to ensure a wide range of tonalities.
- Conversation. This class consisted of thousands of audio clips obtained from podcasts in both Spanish and English, featuring both male and female speakers.

**Figure 9:**
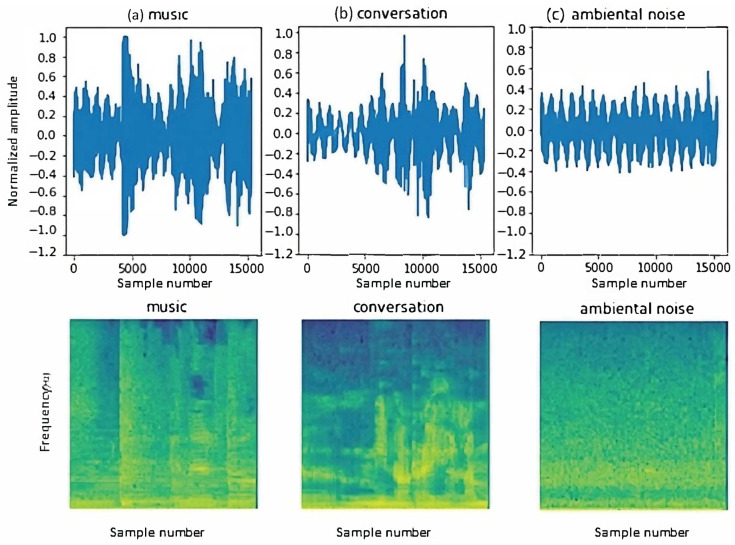
Examples of audio signals for the new classes: (**a**) music, (**b**) conversation, and (**c**) ambient noise. Examples of spectrograms of audio signals for the new classes: (**a**) music, (**b**) conversation, and (**c**) ambient noise.

Two sets of data were generated simultaneously with recordings of the same videos and podcasts, one with 1 s durations and another with 2 s durations. The best results were obtained from the first dataset, so some examples of the samples taken for the new classes are shown in Figure 9.

For the “non-cough” class, audio from the ESC-50 dataset was used, excluding samples corresponding to the cough classes used in the first model mentioned in this document. The new dataset had the same characteristics as the audio used for the previous models:

- 1 s in duration;
- Mono-channel;
- 16 bits;
- 16 kHz bit rate.

The final dataset consisted of 5k audio clips evenly distributed across each class, resulting in 1k audio clips per class. In the detection test performed on the computer, to verify the proper operation of the neural network before migrating the model from TensorFlow to TensorFlow Lite, it was observed that the accuracy rate was greater than 95% in audio that included silence, music, and cough sounds.

As explained in the previous section, once the model was tested in TensorFlow, it was exported to TensorFlow Lite to allow it to function in real time on a Raspberry Pi. As the device is intended for use in noisy environments, background noise was added to assess its impact on performance, in addition to cough sounds and interview audio clips. It is interesting to note that the accuracy dropped to approximately 85–90%. This is due to the conversion of the model from TensorFlow to TensorFlow Lite, which reduces the model’s size and latency in exchange for a slight decrease in accuracy.

## 3. Experimental Results and Interoperability Testing

### 3.1. Full System Integration

All the machine learning environment of the Raspberry Pi was implemented in Python, this allows the system to run in real time, providing a more seamless and efficient operation. Specifically, Python version 3.10 was used due to its robustness and wide range of supported libraries.

The final system’s schematic, as shown in Figure 1, illustrates the various components of the system and their interactions. The functions implemented in the system are as follows:

- Continuous capture and analysis of environmental audio: The system continuously captures environmental audio and analyzes it to detect coughs. Upon detection of a cough, the system generates a JSON packet and sends it via TCP to a server. This packet includes both an audio event alert and the base64-encoded audio for subsequent analysis or validation.
- Temperature monitoring and calibration: The system uses an MLX90640 thermopile sensor for temperature monitoring. For calibration, two Peltiers and two thermometers (BMP085 and MCP9800) are used, as mentioned earlier. This ensures that the temperature readings from the thermopile are accurate and reliable.
- Automatic server address discovery: The system can automatically discover the server address through UDP packets. If there is a server to send data to on the same network, it will send UDP packets that the device will receive to configure the transmission of TCP packets to that IP and port. This feature simplifies the setup process and enhances the system’s usability.

The sensor event transmission is encapsulated in a package that contains metadata to identify the event type, the user generating it, and the specific sensor that triggered it. The first event transmission package has the following configuration:

- Device ID (“id” field): This field is related to the user who generated the event. A dimension table for translating the device name to the associated user name is included. It is created from a common name and the last four digits of the device’s MAC address.
- Timestamp (“time” field): This field identifies the moment when the event occurred in epoch time format. This allows for precise timing of events, which is crucial for real-time applications.
- Type (“Type” field): This field indicates the package’s origin. Initially, only event type packages (“event”) are implemented, but in the future, control and test packages may be implemented. This provides flexibility for future enhancements to the system.
- Sensor: This field can take two values, “thermal” or “cough”. In the case of “thermal”, it refers to the temperature sensor and also includes a “temperature” field with the patient’s temperature at that time. This event is only sent if the patient’s temperature is above 38 °C. The other value it can take is “cough”, indicating the detection of a cough in the audio. Additionally, this event adds an “audio” field to the package containing the audio encoded in base64.

The JSON package would look like this:

“Event”: “id”: “E-1234”, “time”: “event time in UNIX time format”, “type”: “thermal”, “temperature”: “detected temperature”

“Event”: “id”: “E-1234”, “time”: “event time in UNIX time format”, “type”: “cough”, “Audio”: “base64-encoded audio array”

**Figure 10:**
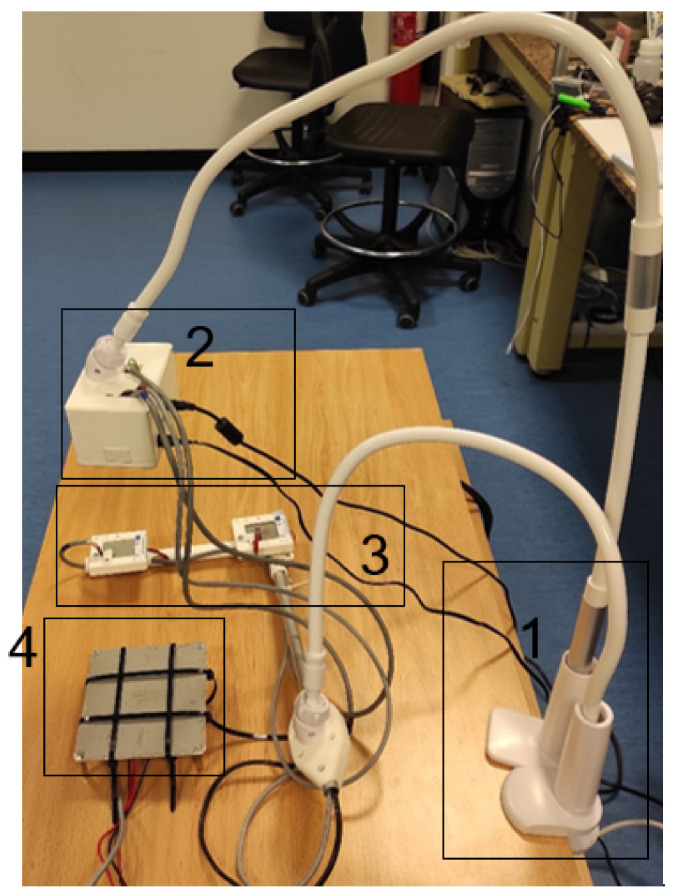
Complete system with validation tool. The different parts of the system are (1) bed mounting; (2) control unit; (3) Peltiers for calibration; (4) reference system for tests.

In Figure 10, the final prototype of the system can be observed. The system consists of several parts: (1) bed mounting, which holds the system in place; (2) control unit, which houses the Raspberry Pi and other electronic components; (3) Peltiers for calibration, which provide reference temperatures for calibrating the thermopile sensor; and (4) reference system for tests, which is used for system validation and testing.

### 3.2. Tests

Once the individual components of the system were tested and validated separately, comprehensive tests were conducted to ensure the correct operation of all components together. This integrated testing approach is crucial for verifying the overall functionality of the system and identifying any potential issues that may arise when the components interact with each other. The tests included temperature monitoring under various conditions. This was to evaluate the system’s performance in different scenarios and ensure that it could accurately measure temperature regardless of the environmental conditions. During this monitoring, both cough and non-cough audio samples were played to verify correct detection (a demonstration video can be found in the Supplementary Material). This allowed us to test the system’s ability to distinguish between cough sounds and other sounds, which is crucial for its intended application. For the complete device test, a constant and cyclic temperature variation was implemented. This involved repeatedly changing the temperature in a controlled manner and observing the system’s response. This test was designed to evaluate the system’s ability to accurately track changes in temperature over time. While the temperature was being varied, audio samples were played simultaneously. This allowed us to test the system’s ability to perform temperature monitoring and cough detection concurrently, which is representative of real-world usage scenarios. Regarding cough detection, different configurations were tested. These ranged from complete silence, where no sound is present, to conversations or coughs with background noise. This was to evaluate the system’s performance in various acoustic environments and ensure that it could accurately detect cough sounds even in the presence of other sounds. The results of the tests were promising. When silence was detected, the system correctly identified it and did not display any false positives. If a cough was detected, the system correctly identified it and displayed “cough”. Any other sound was correctly identified as “other”, demonstrating the system’s ability to distinguish between cough sounds and other sounds.

Temperature detection also performed well in the tests. Despite the complexity of the system and the challenges associated with non-contact temperature measurement, the system was able to maintain accuracy in temperature detection. This is a testament to the effectiveness of the thermal calibration methods and the performance of the infrared radiation sensors used in the system. In conclusion, the tests demonstrated that the system performs well in a variety of conditions and is capable of accurate temperature monitoring and cough detection. These results are encouraging and suggest that the system could be a valuable tool for remote patient diagnostics.

### 3.3. Interoperability Testing

Interoperability testing was performed to ensure that the system could communicate effectively with other systems. This is a crucial aspect of the system’s functionality, as it needs to be able to exchange data with other systems in a reliable and standardized manner.

This involved testing the system’s ability to send and receive data from a server. The system was designed to send sensor event data to a server in the form of JSON packets via TCP. These data include information about the type of event (e.g., a cough detection or a temperature reading), the time of the event, and other relevant details. The server, in turn, was set up to receive these packets and process the contained data. This could involve storing the data in a database, triggering alerts, or performing other actions based on the data. The test involved sending multiple packets from the system to the server and verifying that the server received and correctly interpreted the data. This was achieved by comparing the data sent by the system with the data received and processed by the server. Any discrepancies would indicate a problem with the system’s data transmission capabilities or the server’s data processing capabilities. In addition to testing the system’s ability to send data to a server, the interoperability testing also involved testing the system’s ability to discover the server’s address automatically. This feature is designed to simplify the setup process and enhance the system’s usability. It works by having the system listen for UDP packets from the server, which contain the server’s IP address and port. Upon receiving a UDP packet, the system configures itself to send TCP packets to the server’s IP address and port. The test involved setting up a server to send UDP packets and verifying that the system correctly received these packets and configured itself to send TCP packets to the server’s IP address and port. This was achieved by observing the system’s network traffic and verifying that it was sending TCP packets to the correct address and port. The results of the interoperability tests were positive. The system was able to successfully send data to the server and correctly discover the server’s address. This demonstrates that the system is capable of interoperating with other systems, which is crucial for its intended application in remote patient diagnostics. It shows that the system can be integrated into a larger healthcare infrastructure, where it can contribute valuable data for patient monitoring and disease detection.

## 4. Discussion

Thermal and acoustic sensors have unique advantages in remote health monitoring, which can make them more suitable than other types of sensors in certain applications. In the case of thermal sensors, they can measure body temperature, which is a vital sign for diagnosing numerous diseases. Changes in body temperature can indicate fever, infection, or other health conditions. Thermal sensors can provide continuous, non-invasive monitoring of body temperature, making them ideal for remote health monitoring [19]. Additionally, acoustic sensors can monitor respiratory behavior, another critical signal for diagnosing and monitoring diseases. For example, changes in breathing patterns can indicate conditions like sleep apnea, asthma, or heart failure [20]. Acoustic sensors can capture these changes, providing valuable data for remote health monitoring [21]. Compared to other sensors, thermal and acoustic sensors offer high sensitivity, accuracy, flexibility, and stretchability [22]. However, it is important to note that the choice of sensor depends on the specific requirements of the health monitoring system. Other sensors may be more suitable for different applications or health conditions. On the other hand, some challenges exist with other types of sensors used in remote patient monitoring systems. For instance, wearable devices, which often use a variety of sensors, have been associated with issues such as data quality, interoperability, health equity, and fairness [23]. Additionally, the reliability of data from some remote patient monitoring tools has been questioned, with error margins up to 25 percent reported for some popular fitness wearables [24]. Furthermore, remote patient monitoring systems often rely on technology that not all patients can afford, such as reliable internet connections [25].

Thermal sensors’ compact size and low power consumption are pivotal for their integration into wearable health monitoring devices [19]. For example, a thermal sensor embedded in a wristband or smartwatch can monitor body temperature continuously without causing discomfort or inconvenience to the user [26]. This continuous monitoring is crucial in the early detection of fever or infection, especially in vulnerable populations like the elderly or immunocompromised [19]. The sensor’s minimal power requirement ensures a long battery life, essential for uninterrupted health monitoring [19]. On the other hand, the high sensitivity of acoustic sensors is a key attribute in detecting and analyzing respiratory sounds [27]. In the context of sleep apnea detection, an acoustic sensor can be strategically placed in a bedroom to monitor a patient’s breathing patterns during sleep [28]. Its sensitivity allows it to pick up subtle anomalies in breathing, such as pauses or gasps, which are characteristic of sleep apnea [29]. The sensor’s ability to function effectively in a quiet indoor environment, without requiring direct contact with the patient, makes it an unobtrusive and convenient tool for long-term monitoring [29]. In addition, both thermal and acoustic sensors can be optimized for indoor and outdoor settings, depending on their intended use [30]. For instance, thermal sensors used in outdoor health monitoring devices need to be calibrated to account for ambient temperature variations [30]. Similarly, acoustic sensors used outdoors would require noise-cancellation capabilities to accurately detect and analyze health-related sounds amidst environmental noise [30]. In addition, the versatility of these sensors allows for customization based on specific patient requirements. For patients with chronic conditions requiring constant monitoring, sensors with higher sensitivity and longer battery life can be used [31]. Conversely, for short-term monitoring or less critical applications, more basic sensor models may suffice [32].

In summary, the physical attributes and operational settings of thermal and acoustic sensors greatly influence their effectiveness in various health monitoring scenarios. By carefully considering these factors, remote health monitoring systems can be tailored to meet diverse needs, enhancing patient care and outcomes. While there are studies on cough detection using various neural networks and signal types, none of them utilize FFT (fast Fourier transform) as an input, which is a distinct aspect of our approach. In the realm of thermopile-based technologies, the research is limited, with only a few significant contributions, primarily from conference communications. Our work stands out in combining thermal and acoustic data analysis for diagnostics, a synergy not explored in the existing literature. This innovative combination, along with our unique methodology, contributes significantly to the field and enhances the potential for accurate diagnostics. A comparative study can be found in Table 3.

## 5. Conclusions

The advancements in remote patient diagnostics have opened up new possibilities for healthcare. The development of non-invasive sensor technologies, coupled with the power of artificial intelligence, has led to the creation of systems that can monitor key health indicators such as body temperature and cough sounds.

In this work, we have presented a unique approach to remote health monitoring by integrating thermal and acoustic sensors with artificial intelligence. This integration allows for innovative thermal calibration methods and the analysis of acoustic signals to quantify coughing episodes. The novelty of the research lies in its comprehensive integration of different technologies for enhanced diagnostics in healthcare. The study conducts practical experimentation and interoperability tests, validating the concept for each system component, thus advancing non-invasive sensor technology applications in healthcare. Specifically, we have proposed a system that utilizes an ESP32 and a Raspberry Pi model 3B+ for data collection and preprocessing. The system employs novel methods of thermal calibration and assesses low-cost infrared radiation sensors for facial temperature analysis. It also investigates innovative approaches to analyzing acoustic signals for quantifying coughing episodes.

The system was designed to meet two main specifications: cough detection and temperature measurement. Cough detection is achieved using a microphone and a neural network, while temperature measurement is carried out using a thermopile sensor. The thermopile adjusts its output values through a calibration process, reducing the error within a temperature range defined by the calibration points. The hardware utilized in the study, the ESP32 and the Raspberry Pi model 3B+, offer a range of features that make them suitable for our application. They provide ample computational power, memory, and connectivity options, and operate within a wide range of environmental conditions.

The field of remote patient diagnostics is experiencing rapid advancements, but challenges still need to be addressed to improve the accuracy and reliability of these technologies. Our research integrates diverse data capture technologies to analyze them collectively, considering their temporal evolution and physical attributes. Our aim is to extract statistically significant relationships among various variables for valuable insights.

In conclusion, the integration of non-contact thermal and acoustic sensors, low-cost hardware, and neural networks holds great promise for the future of remote patient diagnostics. With further research and development, these technologies could revolutionize healthcare, making it more accessible and efficient.

## Figures and Tables

**Table 1:** Measured temperature values and their errors.

Measured Temperature	Validation Temperature	Error
38.13 °C	38.5 °C	0.37 °C
38.477 °C	38.56 °C	0.083 °C
38.477 °C	38.5 °C	0.023 °C
38.5 °C	39.25 °C	0.75 °C
38.5 °C	39.25 °C	0.75 °C
37.37 °C	37.81 °C	0.44 °C
37.37 °C	37.81 °C	0.44 °C
37.89 °C	37.75 °C	0.14 °C
38.44 °C	37.81 °C	0.63 °C
37.72 °C	37.38 °C	0.34 °C
36.79 °C	37.38 °C	0.59 °C
37.88 °C	37.44 °C	0.44 °C
37.62 °C	37.44 °C	0.18 °C
37.58 °C	36.94 °C	0.64 °C
37.61 °C	36.88 °C	0.73 °C
37.06 °C	36.94 °C	0.12 °C
37.08 °C	36.94 °C	0.14 °C
37.06 °C	36.75 °C	0.31 °C
36.62 °C	36.69 °C	0.07 °C
37.08 °C	36.75 °C	0.33 °C
37.59 °C	36.69 °C	0.9 °C
36.44 °C	36.83 °C	0.39 °C
36.44 °C	36.69 °C	0.25 °C
36.5 °C	36.65 °C	0.15 °C
36.5 °C	36.75 °C	0.25 °C

**Table 2:** Comparative parameters of the generated deep learning models.

Model	Size	Accuracy (%)
1	3.85 MB	90
2	15 kB	88
3	6.351 kB	94

**Table 3:** Comparison of this work with previous literature.

Article	What It Is About	Differences with Our Work
[33]	Cough detector based on audio and accelerometer signals.	We use the audio signal to detect cough, consistently shown to be more accurate. Additionally, we combine it with temperature detection to achieve a more accurate diagnosis.
[34]	Cough detector based on deep neural network (DNN) and Gaussian mixture model (GMM).	We use another neural network, a convolutional one (CNN).
[35]	Cough detector based on machine learning from an accelerometer attached to the bed signal.	Our work is based on the audio signal as they have been shown to be more accurate.
[36]	Compensate temperature of thermopile in an industrial environment with a PSO-BP algorithm.	Our work used thermal compensation as the temperature compensator because it is more accurate.
[37]	Based on microwave for breath and heart rate detection. This device does not detect cough.	Our work also detects cough, which is a substantial difference because cough is a main identification of illness.
[38]	Thermopile-based fever detector.	Our work achieves better accuracy and also complements it with a cough detector, probed as a good indicator of illness.

## Data Availability

The datasets generated and/or analyzed during the current study are available from the corresponding author upon reasonable request.

## Supplementary Materials

The following supporting information can be downloaded at: https://www.mdpi.com/article/10.3390/s24010129/s1.
