# Supplementary material for: Non-Contact Thermal and Acoustic Sensors with Embedded Artificial Intelligence for Point-of-Care Diagnostics
Source: Sensors (Basel). 2023 Dec 26;24(1):129. doi: 10.3390/s24010129 (PMC10781379; doi:10.3390/s24010129)
Supplement: Supplementary file 1 [file sensors-24-00129-s001.zip › sensors-2750256-supplementary.pdf]

1. FACIAL TEMPERATURE EMULATOR

To characterize the system, a validation system was developed as previously explained in section 2.2. For its calibration, the 11x11 cm square was divided into approximately 9 equal parts, and the temperature at each of these points was measured with the DAQ970A[6]. The following images illustrate the process of measuring the different temperatures:

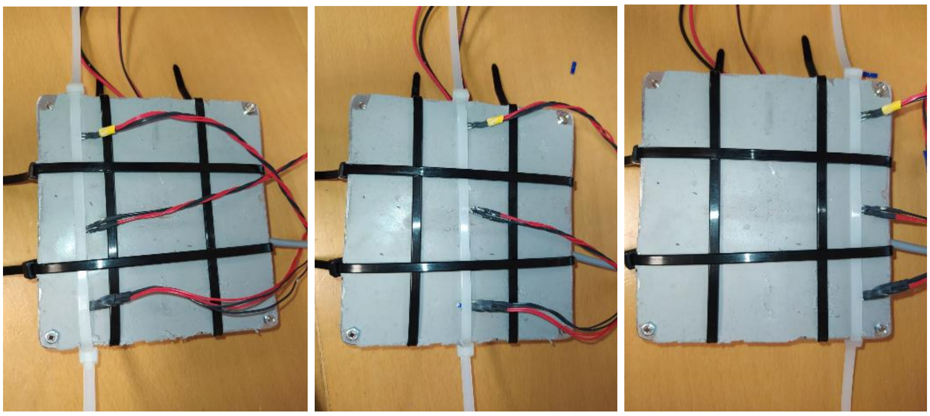

Figura S1. Detail of the position of the measuring probes.

At this point it should be specified that the different measurements taken are shown in the form of a 3x3 table where each point corresponds to one of the sectors of the validation system. The measurements obtained are as follows:

Table S1: Measurements taken at room temperature (24.5 ºC)

24.31	24.37	24.32
24.5	24.51	24.49
24.65	24.62	24.66

Table S2: Measurements taken with temperature set at 36.5 ºC

35.88	36.01	35.93
37.35	38.23	37.33
35.87	35.98	35.9

Table S3: Measurements taken with temperature set at 37ºC

36.63	36.74	36.61
37.74	38.51	37.7
36.64	36.72	36.69

Table S4: Measurements taken with temperature set at 37.5 ºC

37.18	37.35	37.27
38.56	39.41	38.52
37.23	37.48	37.34

Table S5: Measurements taken with temperature set at 38 ºC

37.97	38.06	37.94
39.21	39.87	39.08
38.08	38.21	38.09

Table S6: Measurements taken with temperature set at 38.5ºC

38.36	38.53	38.42
39.43	40.42	39.54
38.34	38.57	38.38

2. Neural networks

Next, we can see the parameters with which the neural network has been designed and the model obtained:

```
Input shape: (129, 124, 1)
Model: "sequential"
_________________________________________________________________
 Layer (type)                Output Shape              Param #
=================================================================
 resizing (Resizing)         (None, 32, 32, 1)         0

 normalization (Normalizatio  (None, 32, 32, 1)        3
 n)

 conv2d (Conv2D)             (None, 30, 30, 32)        320

 conv2d_1 (Conv2D)           (None, 28, 28, 64)        18496

 max_pooling2d (MaxPooling2D  (None, 14, 14, 64)       0
 )

 dropout (Dropout)           (None, 14, 14, 64)        0

 flatten (Flatten)           (None, 12544)             0

 dense (Dense)               (None, 128)               1605760

 dropout_1 (Dropout)         (None, 128)               0

 dense_1 (Dense)             (None, 2)                 258

=================================================================
Total params: 1,624,837
Trainable params: 1,624,834
Non-trainable params: 3
_________________________________________________________________
```

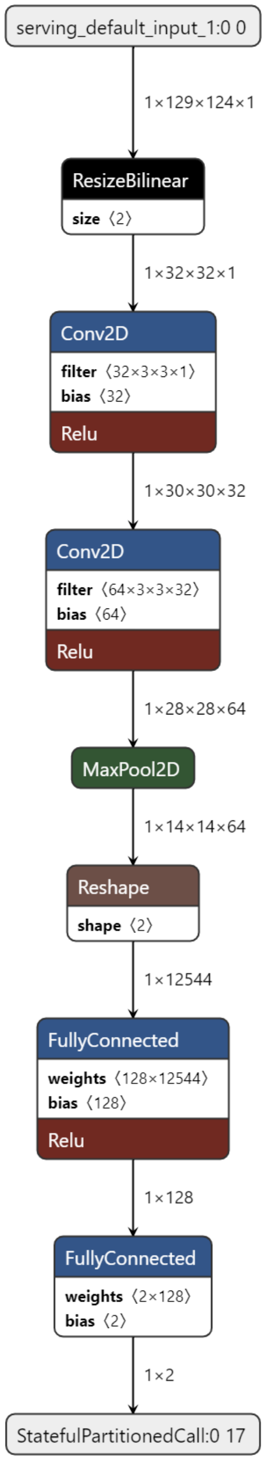

Figure S2. Selected model (left) and diagram of the neural network (right).
